# Deciphering the molecular classification of pediatric sepsis: integrating WGCNA and machine learning-based classification with immune signatures for the development of an advanced diagnostic model

**DOI:** 10.3389/fgene.2024.1294381

**Published:** 2024-01-29

**Authors:** Junming Huang, Jinji Chen, Chengbang Wang, Lichuan Lai, Hua Mi, Shaohua Chen

**Affiliations:** ^1^ Department of Urology, Guangxi Medical University Cancer Hospital, Nanning, Guangxi, China; ^2^ Department of Urology, The First Affiliated Hospital of Guangxi Medical University, Nanning, Guangxi, China; ^3^ Department of Laboratory, The People’s Hospital of Guangxi Zhuang Autonomous Region, Nanning, Guangxi, China

**Keywords:** pediatric sepsis, cuproptosis-related genes (CRGs), cluster analysis, gene regulatory networks, immunology

## Abstract

**Introduction:** Pediatric sepsis (PS) is a life-threatening infection associated with high mortality rates, necessitating a deeper understanding of its underlying pathological mechanisms. Recently discovered programmed cell death induced by copper has been implicated in various medical conditions, but its potential involvement in PS remains largely unexplored.

**Methods:** We first analyzed the expression patterns of cuproptosis-related genes (CRGs) and assessed the immune landscape of PS using the GSE66099 dataset. Subsequently, PS samples were isolated from the same dataset, and consensus clustering was performed based on differentially expressed CRGs. We applied weighted gene co-expression network analysis to identify hub genes associated with PS and cuproptosis.

**Results:** We observed aberrant expression of 27 CRGs and a specific immune landscape in PS samples. Our findings revealed that patients in the GSE66099 dataset could be categorized into two cuproptosis clusters, each characterized by unique immune landscapes and varying functional classifications or enriched pathways. Among the machine learning approaches, Extreme Gradient Boosting demonstrated optimal performance as a diagnostic model for PS.

**Discussion:** Our study provides valuable insights into the molecular mechanisms underlying PS, highlighting the involvement of cuproptosis-related genes and immune cell infiltration.

## Background

Sepsis, characterized by a combination of physiological, pathological, and biochemical abnormalities triggered by an infection., is a severe systemic condition characterized by the presence of bacteria or toxins in the bloodstream. It constitutes a life-threatening infection that requires immediate medical attention (Singer et al., 2016). Recent statistical data highlight sepsis’ varying incidence across regions, yet it remains a prevalent and challenging global concern (Fleischmann et al., 2016a). Notably, sepsis displays a prominent peak in both incidence and mortality within the extremities of the age spectrum, with the highest risk observed among neonates, pediatric patients, and the elderly population (Angus et al., 2001; Fleischmann et al., 2016b; Agyeman et al., 2017). A systematic review underscores a significant rise in incidence among infants (516 cases per 100,000 infants) (Angus et al., 2001). However, this incidence gradually declines as children progress through the 0–19 years age range, reaching 89 cases per 100,000 children. Among children under 5 years old, sepsis and infections have led to 6.3 deaths per 1,000 live births. Once diagnosed, pediatric sepsis (PS) exhibits an estimated case-fatality rate of 25% (Weiss et al., 2015). The pathological mechanisms of PS involve a complex interplay of immune dysregulation, excessive inflammation, release of microbial toxins, microcirculatory dysfunction, and coagulation abnormalities. Infection-induced immune system imbalance triggers a systemic inflammatory response, while the release of microbial toxins and microcirculatory disturbances further exacerbates the condition. Additionally, coagulation abnormalities are common in PS patients, increasing the risk of both thrombosis and bleeding. These factors collectively contribute to the development of multiple organ dysfunction syndrome, highlighting the intricacy and clinical urgency of this condition (Singer et al., 2016; Schlapbach and Kissoon, 2018). Recognizing sepsis’ significant impact, the World Health Organization recently passed a resolution emphasizing sepsis as a major causative factor for preventable morbidity and mortality on a global scale (Singer et al., 2016). Despite significant scientific progress and dedicated efforts, an urgent need persists to enhance our understanding of pathophysiological mechanisms, identify key risk factors, and establish optimal management strategies for PS. By diligently addressing these crucial facets, we aspire to improve treatment outcomes and alleviate the distressing impact of PS on both children and their families.

In recent years, remarkable advancements have been made in comprehensively elucidating programmed cell death mechanisms, given their significant implications in discovering diagnostic biomarkers and therapeutic targets for diverse diseases (Bedoui et al., 2020; Wang et al., 2023). In 2022, Tsvetkov et al. (2022) made a significant discovery in cell biology, unveiling an unprecedented mechanism of cell death induced by copper. Termed “cuproptosis,” this distinct pathway differs from established modes of programmed death, involving direct interactions between copper and lipoylated components within the tricarboxylic acid (TCA) cycle (Supplementary Figure S1). This intricate interaction leads to lipoylated protein aggregation and subsequent depletion of iron-sulfur cluster proteins, culminating in severe proteotoxic stress and cellular demise (Mayr et al., 2014; Solmonson and DeBerardinis, 2018). *FDX1* and protein lipoylation have emerged as pivotal orchestrators during copper ionophore-induced cell death. A significant positive correlation exists between *FDX1* abundance and lipoylated proteins across a range of human tumors (Guo et al., 2023; Zhou et al., 2023). Significantly, copper toxicity has been associated with the interference of iron–sulfur (Fe–S)-containing enzymes. In laboratory settings, copper has demonstrated the inhibition of Fe–S cluster formation by impeding the function of mitochondrial assembly proteins, thereby worsening copper-induced toxicity. Ongoing research suggests that cuproptosis mechanisms will affect multiple diseases, including cancers, Alzheimer’s disease (Lai et al., 2022), heart failure (Yuan et al., 2022a), and Crohn’s disease (Yuan et al., 2022c). Song et al. has elucidated the enigmatic connections between cuproptosis and the pathogenesis of sepsis-induced cardiomyopathy (Song et al., 2023). However, the relationship between cuproptosis-related genes (CRGs) and PS remains unexplored. Thus, unveiling the molecular categorization and genomic heterogeneity of PS cohorts, with a specific focus on cuproptosis and its associated driver genes, holds great importance in significantly advancing our comprehension of pivotal pathogenic mechanisms underlying PS progression and onset.

In our study, our investigation began by examining the expression profiles of CRGs to discern differential expression between individuals afflicted by PS and healthy controls (HC). Subsequently, we delved deeper into immune cell infiltration in these samples. Further, we isolated PS samples from the training set to perform consensus clustering based on the aforementioned differentially expressed CRGs. Our findings revealed the ability to classify patients into two distinct clusters associated with cuproptosis, each exhibiting disparate immune landscapes, functional classifications, and enriched pathways. Employing the Weighted Gene Co-expression Network Analysis (WGCNA) algorithm, we successfully identified pivotal genes linked to cuproptosis clusters, which we intersected with genes associated with PS, uncovering common genes shared between module-related genes in PS and cuproptosis clusters. Subsequently, by comparing various machine learning approaches, we developed a diagnostic model for PS. To validate the model’s discrimination capabilities and stability, we evaluated its performance using quantitative real-time polymerase chain reaction (qRT-PCR) of peripheral blood from both PS patients and HC, along with a nomogram, calibration plot, decision curve analysis (DCA), and three independent validation datasets.

## Materials and methods

### Data acquisition and sample information

We obtained four raw datasets from the Gene Expression Omnibus database (https://www.ncbi.nlm.nih.gov/geo/). Using the GSE66099 RNA-seq dataset, consisting of whole blood samples from 229 patients with PS and 47 HC, we developed a diagnostic model for PS. To assess the model’s predictive capacity, independent validation datasets GSE13904, GSE26378, and GSE26440 were utilized. These datasets encompass transcription profiles derived from whole blood samples of both PS patients and HC individuals. Table 1 presents detailed dataset information.

**TABLE 1:** Detailed information on datasets used in the study.

Dataset	Platform	Sample size	Sample species	Sample organism	Mean age of HC (year)	Mean age of PS (year)
GSE13904	GPL570	18 HC and 185 PS	*Homo sapiens*	Whole blood	-	-
GSE26378	GPL570	21 HC and 82 PS	*Homo sapiens*	Whole blood	3.9	3.7
GSE26440	GPL570	32 HC and 98 PS	*Homo sapiens*	Whole blood	2.4	3.4
GSE66099	GPL570	47 HC and 229 PS	*Homo sapiens*	Whole blood	-	-

HC, healthy control; PS, pediatric sepsis.

### Identification and analysis of differentially expressed CRGs

We compiled a collection of 59 CRGs by reviewing previous researches related to “cuproptosis” and conducting a comprehensive search for “cuproptosis” with a relevance score threshold of 1.4, in the GeneCards database (https://www.genecards.org/). Subsequently, the R package “limma” (Ritchie et al., 2015) was employed to perform differential expression analysis of CRGs between PS patients and HC samples. Results were visualized using the R packages “ggpubr” (https://cran.r-project.org/web/packages/ggpubr/index.html) and “pheatmap” (https://cran.r-project.org/web/packages/pheatmap/index.html). A correlation visualization of heterogeneous CRGs was generated using the “circlize” (Gu et al., 2014) package.

### Consensus clustering and cuproptosis patterns in the training set

The training set of PS samples underwent clustering analysis using the “ConsensusClusterPlus” (Wilkerson and Hayes, 2010) package based on CRG expression levels. Consensus matrix plots, consensus cumulative distribution function (CDF) plots, and trace plots were assessed to determine optimal clustering numbers. Principal component analysis (PCA) was employed to visually depict the distribution of cuproptosis-related patterns in samples, focusing on the first two principal components post-clustering.

### Gene set variation analysis (GSVA)

Using the R package “GSVA,” an enrichment analysis was conducted to explore biological processes and pathways associated with different clusters (Hänzelmann et al., 2013). Two gene sets, “c5.go.symbols” and “c2.cp. Kegg.v7.2.symbols,” were obtained from the Molecular Signatures Database. (https://www.gsea-msigdb.org/gsea/msigdb). Significant terms determined by a Student’s *t*-test (*p* < 0.05), were represented in a barplot, displaying upregulated pathways in blue and downregulated pathways in purple.

### Identification of hub genes via WGCNA analysis of gene modules and disease traits

The WGCNA technique was employed to investigate interconnections between gene modules and disease traits, identifying hub genes closely associated with PS pathogenesis. The process involved extracting genes exhibiting the highest 25% variance from the GSE66099 dataset, hierarchical clustering of PS samples, and Pearson’s correlation coefficient computation to construct a similarity matrix. This matrix was then transformed into an adjacency matrix using an appropriate soft threshold power, followed by a topological overlap matrix. A dynamic tree-cutting algorithm clustered genes into modules, with hub genes defined by gene significance (GS) > 0.2 and module membership (MM) > 0.6. The minimum module size threshold was set at 100 genes.

### Analysis of immune cell infiltration

The “CIBERSORT” R package (https://github.com/Moonerss/CIBERSORT), a widely used analytical tool, relies on a reference gene expression signature matrix to estimate the relative proportions of specific cell types within a mixture. Utilizing linear support vector regression, a robust machine learning approach resistant to noise, “CIBERSORT” involves a feature selection process where genes from the signature matrix are adaptively chosen for effective deconvolution of the given mixture. Subsequently, an empirically determined overall P-value is calculated for the deconvolution process (Newman et al., 2015). This analytical tool is employed to determine the relative composition of 22 immune cells based on their expression profiles in the analysis of GSE66099 samples. Subsequently, we examined the relative compositions of these immune cell types across different groups and their correlations with CRGs. The “ggplot2” (https://cran.r-project.org/web/packages/ggplot2/index.html) and “ggpubr” packages were used for visualization.

### Establishment and validation of the diagnostic model for PS using various machine learning algorithms

Following the intersection analysis of genes within the most significant modules using WGCNA, we identified key genes with specific diagnostic potential for PS. To determine the importance of these genes, we employed four machine learning algorithms. The “kernlab,” (https://cran.r-project.org/web/packages/kernlab/index.html) “randomForest,” (https://cran.r-project.org/web/packages/randomForest/) and “xgboost” (https://cran.r-project.org/web/packages/xgboost/index.html) R packages were utilized for this purpose. The “kernlab” package provides functions for Support Vector Machines (SVM), a supervised learning algorithm used for classification and regression analysis. The “randomForest” package implements the Random Forest algorithm, an ensemble learning method that constructs a multitude of decision trees during training and outputs the class that is the mode of the classification or mean regression of the individual trees. The “xgboost” package implements the XGBoost algorithm, which is an efficient and scalable implementation of gradient boosting. In our approach, the disease phenotype served as the response variable, while the genes identified through WGCNA were utilized as explanatory variables. Model construction using the machine learning algorithms was facilitated by the “caret” (https://cran.r-project.org/web/packages/caret/index.html) R package. Subsequently, we performed an exploratory analysis of the model using the “DALEX” (https://cran.r-project.org/web/packages/DALEX/index.html) R package’s explain function. This analysis involved generating cumulative residual distribution maps and residual boxplots using the plot function, aiding in the identification of the optimal diagnostic model. Model performance evaluation was conducted using the “pROC” (https://cran.r-project.org/web/packages/pROC/index.html) package. Further analysis was carried out by selecting the five most important features within the model. Additionally, the diagnostic model’s validation was performed across multiple validation sets.

### qRT-PCR

Whole blood samples were collected from 8 HC and 8 PS patients. Total DNA extraction from the samples was achieved using the FastPure Blood DNA Isolation Mini Kit V2(Sangon, Shanghai, CN). Start-up reagent: FastStart Essential DNA Green Master (Roche, ShangHai, CN). We then utilized the LightCycler^®^ 96 Instrument (Roche Diagnostics Gmbh, Switzerland) to carry out PCR. For internal control purposes, the β-Actin primer pair was utilized. The experimental protocol received ethical approval from the Ethics Committee of the First Affiliated Hospital of Guangxi Medical University. Informed consent was obtained from the legal guardians of each pediatric patient involved in the study. Clinical information for HC and PS patients used in the qRT-PCR is detailed in Supplementary Table S1. Primer sequences are detailed in Supplementary Table S2.

### Enzyme-linked immunosorbent assay (ELISA)

Serum protein levels were determined using human ELISA kits following the manufacturer’s instructions. This double antibody sandwich method ELISA kit facilitated the measurement of serum protein concentrations. Accurate quantification was achieved by measuring the absorbance (OD value) of the yellow-colored solution at 450 nm using a microplate reader. The concentrations of human serum proteins in the samples were calculated using a standard curve. All the reagents used for ELISA were sourced from mlbio (Shanghai, China) and were listed in Supplementary Table S3.

### Statistical analysis

The nonparametric Wilcoxon test was used to compare two groups of data that did not follow a normal distribution, while the Student’s t-test was used for normally distributed data. To explore correlations, a Spearman correlation test was performed. All statistical analyses were conducted using R software version 4.2.3, considering *p* < 0.05 as indicative of statistical significance.

## Results

### Identification of various expression patterns of CRGs in PS

**FIGURE 1:**
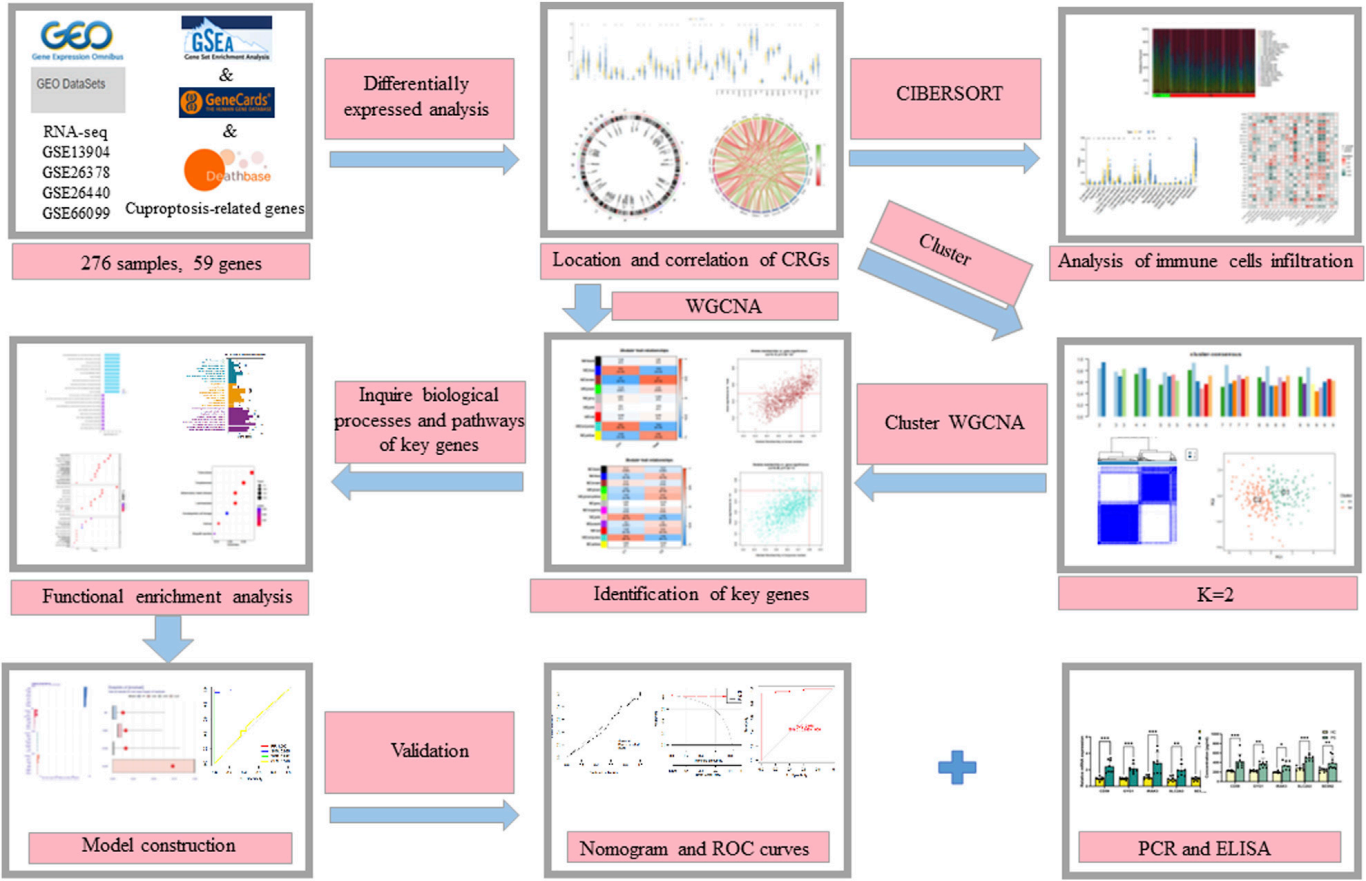
The flow chart of the study.

**FIGURE 2:**
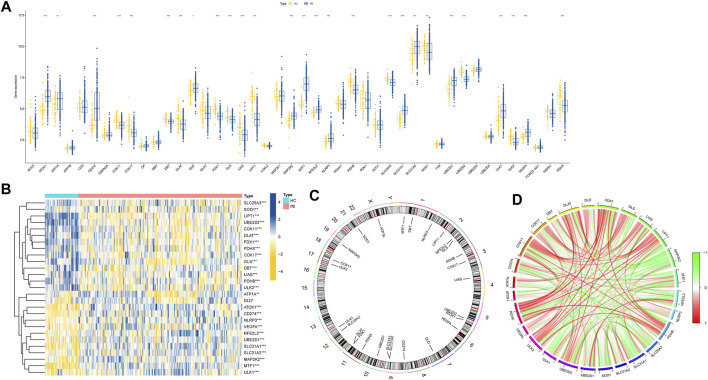
Expression patterns of CRGs in PS. **(A)** Boxplot illustrating the differential expression of 27 CRGs between HC and PS samples. *p < 0.05, **p < 0.01, ***p < 0.001. **(B)** Heatmap displaying relative expression levels of 27 differentially expressed CRGs, *p < 0.05, **p < 0.01, ***p < 0.001. **(C)** Chromosomal locations of the 27 differentially expressed CRGs. **(D)** Correlation analysis demonstrates relationships among the 27 differentially expressed CRGs, where positive and negative correlations are indicated by red and green lines, respectively.

The flow chart of the study is shown in Figure 1. We initiated our study by compiling a collection of 59 CRGs obtained from public databases, as described earlier. The role of 59 CRGs in the cuproptosis pathway are listed in Supplementary Table S4. To explore the expression patterns of these CRGs in PS, we analyzed gene expression data from 229 whole blood samples from PS patients and 47 samples from HC within the GSE66099 dataset. Following logarithmic transformation, we made an intriguing discovery: among the 59 CRGs, 27 were detectable and exhibited differential expression in the PS samples. Specifically, we observed significantly elevated expression levels of *ATP7A*, *DLD*, *ATOX1*, *CD274*, *NLRP3*, *VEGFA*, *NFE2L2*, *UBE2D1*, *SLC31A1*, *SLC31A2*, *MAP2K2*, *MTF1*, and *ULK1* in PS samples. Conversely, reduced expression levels were observed for *SLC25A3*, *SOD1*, *LIPT1*, *UBE2D2*, *COX11*, *DLAT*, *FDX1*, *PDHX*, *COX17*, *GLS*, *DBT*, *LIAS*, *PDHB*, and *ULK2* in the PS samples (Figure 2A). A heatmap was generated to visually represent the divergent expression of these differentially expressed CRGs between PS patients and HC individual whole blood samples (Figure 2B). Additionally, Figure 2C illustrates the chromosomal locations of these CRGs, while Figure 2D reveals varying degrees of correlation among the differentially expressed CRGs (Figure 2D), suggesting potential interactive regulatory relationships in PS.

The significance of innate and adaptive immune responses in PS progression has gained increasing recognition. Distinct variations are observed in pivotal immune cells within neonatal immune responses. Notably, children exhibit reduced levels of neutrophils, antigen-presenting cells, monocytes, and dendritic cells (DC) (Willems et al., 2009; Mantovani et al., 2011), rendering immune cells promising therapeutic targets. In our study, the CIBERSORT algorithm was utilized to determine relative immune cell abundances within GSE66099 dataset samples, with visualization via a heatmap (Supplementary Figures S2A). Subsequent correlation analysis, depicted in Supplementary Figures S2B, revealed close associations between differentially expressed CRGs and distinct immune cell populations in the local environment. Striking correlations were observed, particularly between naive CD4^+^ T cells and CD8^+^ T cells, strongly linked to 24 immune cell subtypes. This underscores the concurrent presence of CRGs and specific immune cell subpopulations within the local blood environment. Such correlation potentially contributes to understanding the link between cuproptosis and PS onset, progression, and treatment responsiveness. Importantly, varying proportions of infiltrating immune cell types were evident across different cohorts (Supplementary Figures S2C). In summary, our findings emphasize the pivotal role of CRGs in PS development and their profound impact on the immune microenvironment.

### Unsupervised clustering analysis of differentially expressed CRGs in PS samples and machine learning algorithm

**FIGURE 3:**
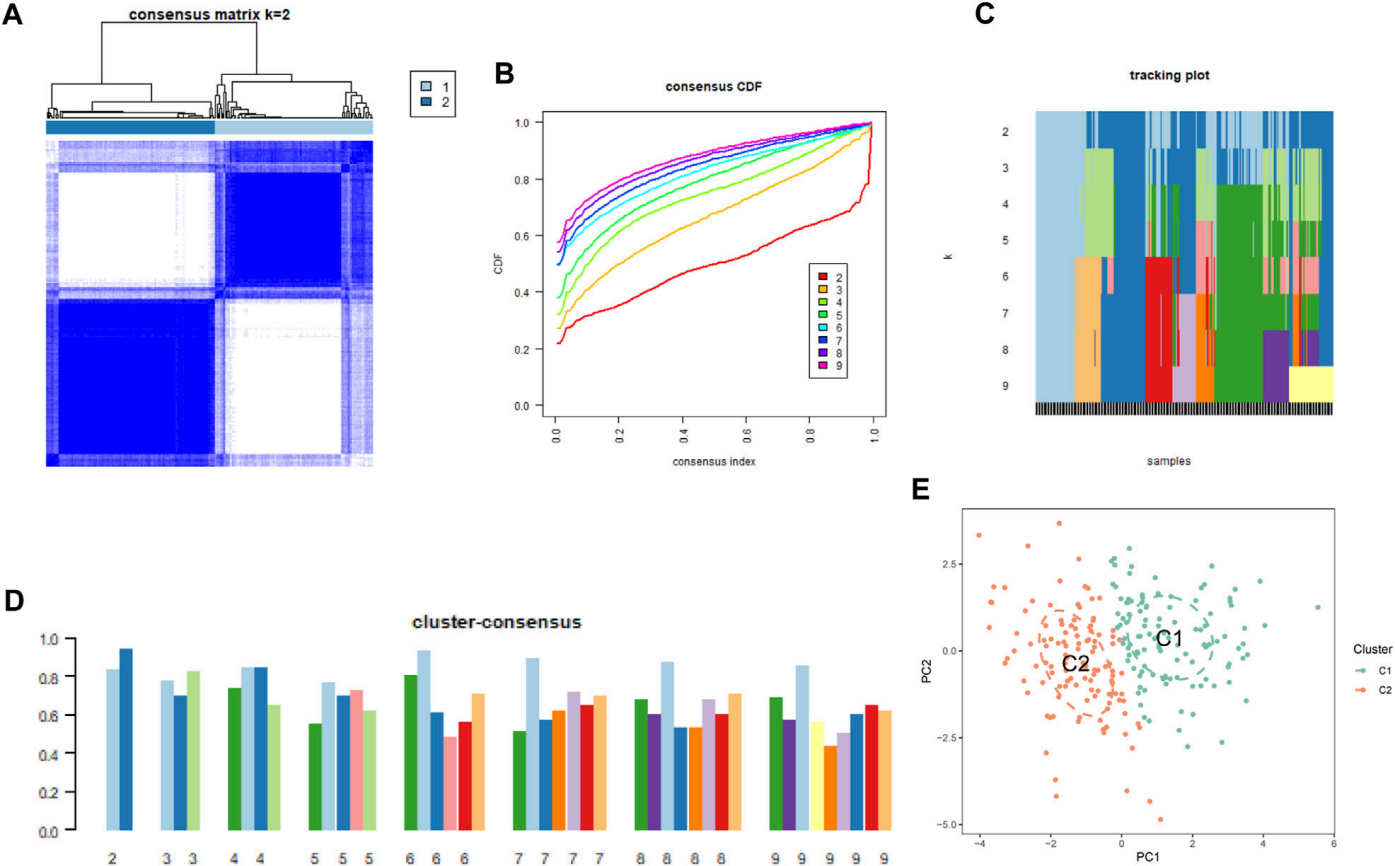
Cluster analysis of differentially expressed CRGs in PS samples. **(A)** Samples were divided into 2 distinct clusters when k = 2. **(B)** Consensus clustering CDF for k = 2 to 9. **(C)** Trace plot displaying clustering results for each sample at varying k values. (2–9) **(D)** Consensus clustering scores are computed when k values are systematically varied from 2 to 9. **(E)** PCA analysis visually represents the distribution of two identified unsupervised consensus clustering cuproptosis clusters.

To understand the diverse expression patterns of CRGs in PS, we selected a subset of 229 PS samples from the training set. Our analysis using the consensus clustering approach revealed two distinct clusters as the most optimal outcome, as evident from the consensus matrix plots (k = 2) (Figure 3A). The stability of clustering was confirmed by minimal fluctuations in consensus CDF curves at different consensus indexes (Figure 3B), and the trace plot also demonstrated the stability of the clustering (Figure 3C). Moreover, the consistency score for each cluster exceeded 0.8, when k = 2 (Figure 3D). Consequently, we divided the 229 PS samples into two clusters: Cluster 1 (C1) consisting of 111 samples, and Cluster 2 (C2) comprising 118 samples. Subsequent PCA distinctly demarcated these clusters (Figure 3E).

**FIGURE 4:**
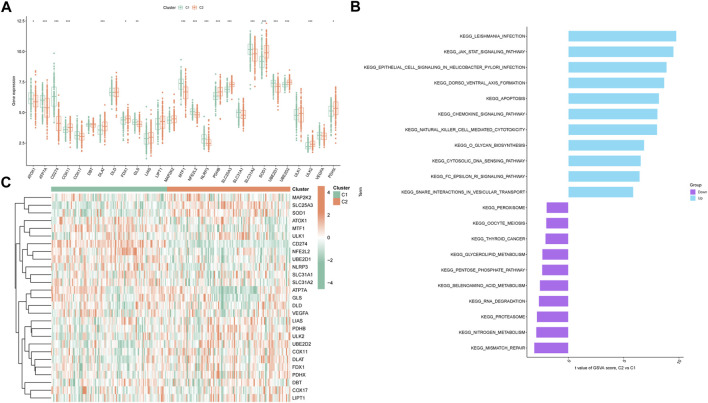
Expression patterns of CRGs in two unsupervised consensus clustering identified cuproptosis clusters. **(A)** Boxplots showing differentially expressed CRGs between two ceproptosis clusters. **(B)** Heatmap showing relative expression levels of 27 CRGs within cuproptosis clusters C1 and C2. **(C)** GSVA enrichment analysis based on the HALLMARK pathway between cuproptosis clusters C1 and C2 samples, ranked by t-value. *p < 0.05, **p < 0.01, ***p < 0.001.

In pursuit of comprehensive insight into molecular characteristics within the distinct cuproptosis clusters, we conducted systematic analyses. Differential expression of multiple CRGs between C1 and C2 was observed, with 18 out of 27 CRGs displaying differential expression (Figure 4A). A heatmap effectively depicted the relative expression patterns of these 27 CRGs in PS samples (Figure 4B). Moreover, GSVA highlighted the upregulation of infectious-related pathways and immune signaling pathways in C2, including leishmania infection, JAK-STAT signaling pathway, chemokine signaling pathway, and natural killer cell-mediated cytotoxicity. Conversely, C1 exhibited enrichment in metabolism-related pathways such as nitrogen metabolism and selenoamino acid metabolism (Figure 4C). Furthermore, the CIBERSORT algorithm was utilized to estimate the proportions of infiltrating immune cells in the two clusters. The barplot displayed relative immune cell abundances (Supplementary Figures S3A), while the boxplot illustrated comparisons of various infiltrating immune cell types (Supplementary Figures S3B). Strikingly, significant differences in relative abundance were observed among 12 infiltrating immune cell types. This comprehensive analysis provided detailed insights into differences between the two cuproptosis clusters, to gain further insights into their underlying mechanisms.

### Application of WGCNA for identification of key genes linked to PS and cuproptosis

**FIGURE 5:**
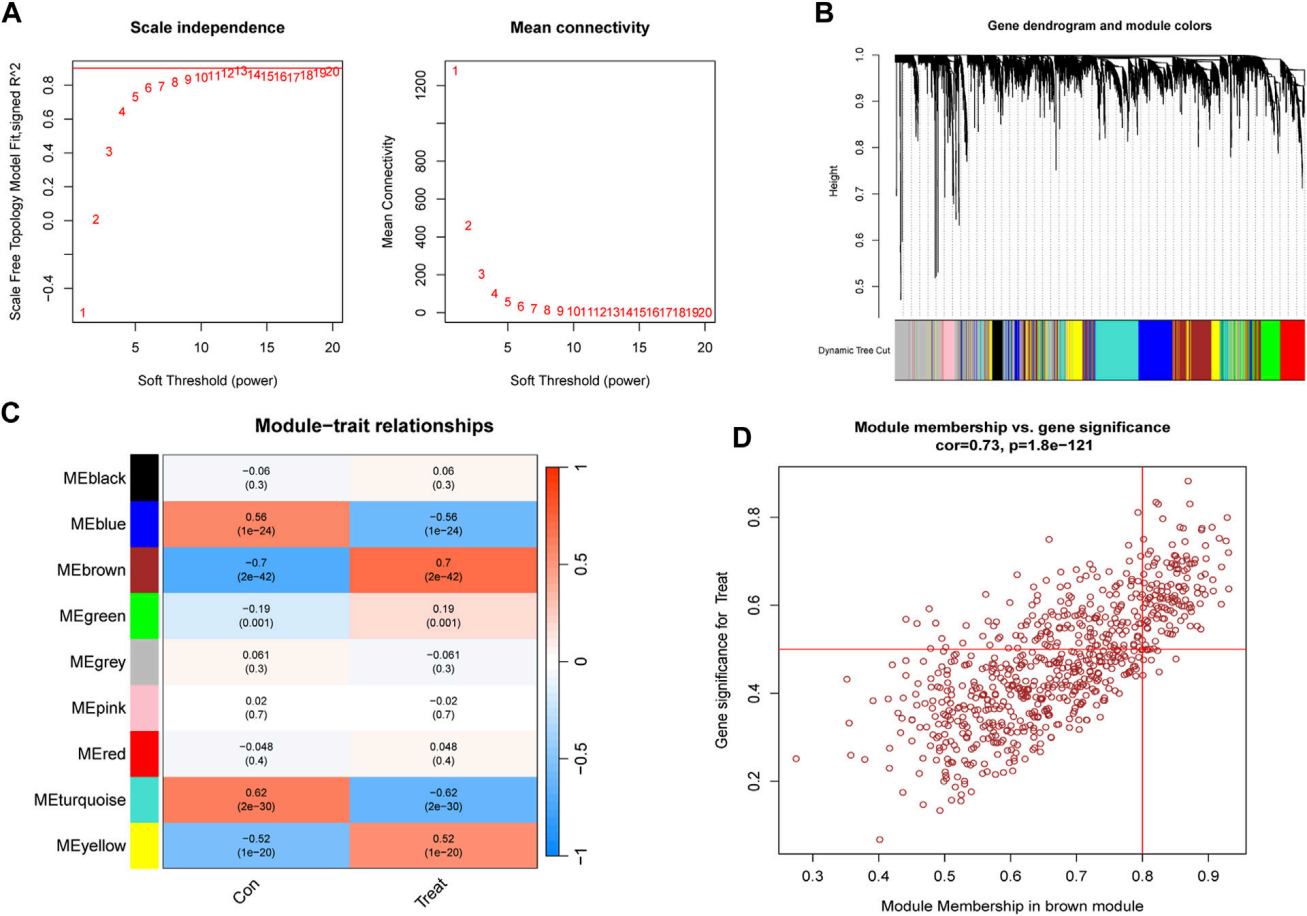
Construction and module analysis of WGCNA. **(A)** Network topology analysis conducted by varying soft-threshold powers. **(B)** Clustering dendrogram illustrating hierarchical gene grouping by topological overlap, with assigned module color indicating different gene clusters. **(C)** Correlation analysis examining relationships between distinct co-expression modules and clinical traits. **(D)** Relevance of brown module members to PS.

To identify key genes associated with PS, we used the WGCNA algorithm. After selecting genes with the top 25% variance and excluding abnormal samples from the GSE66099 dataset, a scale-free network was established with a soft threshold of 11, resulting in a scale-free *R* ^2^ value of 0.86 (Figure 5A). This approach identified nine distinct co-expression modules (Figure 5B). Notably, the brown module exhibited the highest correlation (r = 0.7) and significant *p*-value (P = 2e^−42^) with PS (Figure 5C). Subsequently, 509 genes within this module were subjected to further analysis, revealing a significant positive correlation between the brown module and corresponding genes (Figure 5D).

Subsequently, we aimed to identify key genes associated with cuproptosis clusters in PS subjects in the GSE66099 dataset using the WGCNA algorithm. Using a soft threshold of β = 8 and an *R* ^2^ value of 0.89, we successfully established a scale-free network (Supplementary Figures S4A, B). Once again, the turquoise module demonstrated the highest correlation (r = 0.54) and significant *p*-value (P = 4e^−19^) with cuproptosis clusters (Supplementary Figures S4C). Consequently, 480 genes within this module were selected for further analysis, revealing a significant correlation with the module (Supplementary Figures S4D).

An intersectional analysis was performed on the key genes obtained through WGCNA, revealing 231 shared genes between module-related genes in PS patients and HC individuals, as well as module-related genes in cuproptosis clusters (Supplementary Figures S5A). Further Gene Ontology (GO) functional enrichment analysis revealed the primary involvement of shared genes in immune receptor activity, oxidative stress signaling pathways, and regulation of the inflammatory response. This emphasized their vital role in mitigating inflammation progression, involving diverse immune cells, factors, and oxidative stress responses within the human body (Supplementary Figures S5B). Similarly, the Kyoto Encyclopedia of Genes and Genomes (KEGG) pathway signaling enrichment analysis highlighted the participation of shared genes in pathways such as MAPK signaling, Toll-like receptor signaling, and TNF signaling (Supplementary Figures S5C).

### Construction of diagnostic model for PS using diverse machine learning approaches

To identify hub genes with diagnostic potential for PS among the 231 shared genes obtained from WGCNA, we employed four machine-learning approaches (RF, SVM, XGB, and GLM) to construct diagnostic models using 70% of the GSE66099 dataset samples. Cumulative residual distribution maps (Supplementary Figures S6A) and residual boxplots (Supplementary Figures S6B) of these four algorithms revealed that XGB and SVM displayed smaller residual values, indicating model reliability. Furthermore, Supplementary Figures S6C shows the top ten variables ranked by root mean square error (RMSE) for each model. The diagnostic performance of these models was assessed using receiver operating characteristic (ROC) curves with the remaining 30% of GSE66099 samples (Supplementary Figures S6D). Impressively, three models exhibited excellent discrimination capabilities, with area under curve (AUC) > 0.99. Based on predictive ability and reliability, XGB emerged as the optimal diagnostic model for PS. The five most important variables in the model, *IRAK3*, *SESN2*, *CD59*, *SLC2A3*, and *GYG1*, were identified as hub genes for further analysis.

Moreover, a nomogram based on these hub genes (*IRAK3*, *SESN2*, *CD59*, *SLC2A3*, and *GYG1*) was constructed (Supplementary FigureS S7A). The calibration plot demonstrated good predictive accuracy between actual and predicted probabilities (Supplementary Figures S7B), and the DCA confirmed the model’s clinical significance in decision-making (Supplementary Figures S7C). In summary, our utilization of machine learning algorithms yielded a diagnostic model for PS using shared genes from WGCNA, with ROC analysis, calibration plots, and DCA, collectively indicating its promising predictive efficiency for PS.

### Validation of the 5-gene diagnostic model using independent external cohorts

Next, we assessed the predictive potential of the 5-gene diagnostic model using independent validation datasets, GSE13904, GSE26378, and GSE26440. ROC analyses consistently exhibited high AUC values: 0.985 in GSE13904 (Supplementary Figures S8A), 1.000 in GSE26378 (Supplementary Figures S8B), and 0.981 in GSE26440 (Supplementary Figures S8C). These results unequivocally demonstrated the excellent discrimination performance and stability of our constructed model, highlighting its promising clinical value. To demonstrate the absence of age bias in the datasets included in the article, we performed a correlation analysis between genes and age for the validation set GSE26378. The results indicate that there is no statistically significant correlation, with an R value consistently below 0.5 (Supplementary Figure S9).

### Verification of hub genes in peripheral blood

**FIGURE 6:**
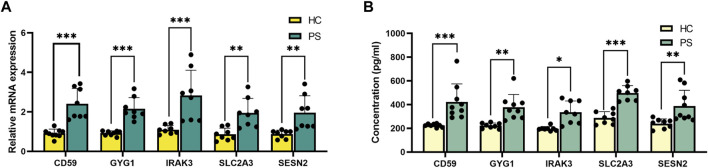
Validation of the hub genes in peripheral blood. **(A)** qRT-PCR verification of *IRAK3*, *SESN2*, *CD59*, *SLC2A3,* and *GYG1* expression between PS patients and HC individuals. **(B)** ELISA verification of IRAK3, SESN2, CD59, SLC2A3, and GYG1 protein levels in serum compared with HC individuals. *p < 0.05, **p < 0.01, ***p < 0.001.

qRT-PCR was conducted to validate the differential expression of hub genes (*IRAK3, SESN2, CD59, SLC2A3, and GYG1*) in peripheral blood samples from PS patients and HC individuals (Figure 6A), consistent with RNA sequencing findings.

ELISA validation experiment assessed protein concentrations of IRAK3, SESN2, CD59, SLC2A3, and GYG1 in serum samples from PS patients and HC individuals (Figure 6B). Notably, significant differences were observed: IRAK3 (335.1 ± 95.6 *versus* 196.7 ± 18.2 pg/mL, *p* = 0.010, SESN2 (387.4 ± 132.9 *versus* 239.9 ± 40.0 pg/mL, *p* = 0.004), CD59 (422.8 ± 151.6 *versus* 224.4 ± 14.5 pg/mL, *p* < 0.001), SLC2A3 (497.4 ± 62.0 *versus* 287.9 ± 52.2 pg/mL, *p* < 0.001), and GYG1 (377.9 ± 107.6 *versus* 222.0 ± 22.5 pg/mL, *p* = 0.002) using rank tests.

## Discussion

Throughout history, the concept of sepsis has evolved from its early microbiological theories, pioneered by figures like Semmelweis, the renowned Hungarian physician who discovered the pathogen responsible for puerperal fever, and Pasteur, to our contemporary understanding as a systemic infection (Paul et al., 2014; Shankar-Hari et al., 2016). Over time, extensive investigations from pathological, clinical, and biological viewpoints have enhanced our comprehension of sepsis pathophysiology (Weiss et al., 2014; Balamuth et al., 2016). The most recent definition, commonly known as sepsis 3.0, characterizes sepsis as a severe, life-threatening condition marked by organ dysfunction due to a dysregulated response to infection (Seymour et al., 2016; Matics and Sanchez-Pinto, 2017). Despite significant advancements in medical knowledge and clinical care, including the Sequential Organ Failure Assessment (SOFA) score for assessing organ dysfunction (Singer et al., 2016), our current scoring systems, including SOFA, have not adequately accounted for the variability in the sub-score criteria (Jhang et al., 2014; Sanchez-Pinto and Khemani, 2016). This complexity underscores that our grasp of sepsis remains intricate and continually evolving. The heterogeneity of sepsis further contributes to diverse phenotypes and treatment responses, posing challenges to clinical outcomes (Seymour et al., 2019; Kellum et al., 2022). Despite ongoing research, understanding underlying mechanisms, identifying PS subtypes, and developing targeted interventions are pivotal for enhancing patient outcomes. Therefore, addressing knowledge gaps and classifying PS subtypes is an essential research and clinical priority.

Our study exploits a critical gap in the literature, offering a comprehensive and systematic exploration of cuproptosis transcriptomic profiles between PS patients and HC individuals. This investigation unmasked significant aberrations in CRG expression patterns within the realm of PS, affirming the profound interplay between cuproptosis and disease pathogenesis. Intriguingly, our analysis unveils distinct immunological landscapes within the PS microenvironment, particularly highlighting heterogeneous subtypes of T cells. These findings exhibited a marked proclivity for significant heterogeneity when compared with HC individuals, and unequivocally underscore T cells’ intimate involvement in PS progression. Specifically, PS samples exhibit altered relative T cell abundances, notably increased T follicular helper cells (Taylor et al., 2020), monocytes (Na et al., 2020), M0 macrophages (Yin et al., 2021), and neutrophils (Qi et al., 2021; Wang et al., 2021), while CD8^+^ T cells (Xie et al., 2019) and DCs(Efron and Moldawer, 2003) are more abundant in HC individuals. These trends were also manifest distinctly in animal models. As frontline immune defenders, macrophages play a pivotal role in PS pathophysiological (Yang et al., 2014). Their function significantly influences septic patient prognosis, with metabolic states directly influencing their immune functions (Karakike and Giamarellos-Bourboulis, 2019). Yuan et al. provide compelling evidence of KLF14’s essential role in modulating macrophage immune function by repressing HK2 transcription during sepsis (McConnell and Yang, 2010), affecting glycolysis and impacting septic mice (Yuan et al., 2022b). Moreover, our identification of two distinct cuproptosis clusters in PS patients using consensus clustering revealed unique innate immunological milieus, particularly involving T cells. Existing studies emphasize T cell infiltration into the brain in septic mice during the acute phase. Inhibiting T cell migration into the brain through the administration of *FTY720*, yielded a sustained manifestation of anxiety-like behavior in septic mice. This outcome cogently illustrates the pivotal role y T cell infiltration plays in the eventual convalescence from sepsis-associated encephalopathy and the concurrent amelioration of mental impairments, particularly during the chronic phase (Saito et al., 2021). These observations collectively signify a highly intricate interplay facilitated by diverse immune cells within the microenvironment, with innate and adaptive immunity assuming the role of conductors. This phenomenon establishes a mechanistic connection between aberrant immune responses in the immune microenvironment and the disease manifestations of PS.

Recent times have witnessed remarkable progress in diagnosing and treating diseases like PS through the integration of machine learning algorithms with various phenotypes, including clinical test results (Qin et al., 2022). This approach presents a valuable opportunity to decipher the disease’s heterogeneity and implement precise classifications, thereby significantly to the advancement of precision medicine. Descriptive evidence notably suggests that machine learning algorithms surpass traditional clinical approaches in predicting severe PS (Le et al., 2019; Banerjee et al., 2021), defining PS subgroups (Qin et al., 2023), and enabling early personalized anti-inflammatory clinical treatments with improved accuracy and efficiency in diagnosing PS (Qin et al., 2022). However, previous studies in this field have been constrained by limitations such as sample sizes, restricted validation cohorts, and reliance on a single learning algorithm. Consequently, the reliability of constructed diagnostic models has come under scrutiny, challenging the original aspirations of researchers. In response to these limitations, our present study is dedicated to constructing a robust diagnostic model for PS by employing four distinct machine-learning algorithms. Notably, among these algorithms, XGB has emerged as the most stable and accurate performer, surpassing the others in predictive power. To gain deeper insights, we identified the five most important variables, which function as hub genes: *IRAK3*, *SESN2*, *CD59*, *SLC2A3*, and *GYG1*. These hub genes were subjected to further investigation to elucidate their essential roles in PS development. To validate our findings, qRT-PCR analyses were conducted on peripheral blood samples collected from both PS patients and healthy individuals. The remarkable outcome is that the 5-gene diagnostic model displayed excellent discrimination performance and stability when subjected to testing against three independent validation datasets. These findings not only confirm the promising clinical value of the constructed model but also highlight the crucial functions of the identified hub genes in the pathogenesis of PS.

The gene *IRAK3*, encoding interleukin-1 receptor-associated kinase 3, plays a key role in immune response regulation (Nguyen et al., 2022a). Sepsis is characterized by two distinct phases: an initial hyper-inflammatory phase characterized by a remarkable surge in potent cytokines like TNF-α and IL-6, followed by an immunosuppression phase wherein inflammatory cytokines levels significantly decrease (Hotchkiss et al., 2013). Meta-analyses of *in vivo* studies substantiate the roles of *IRAK3* during the immunosuppression phase of sepsis (Nguyen et al., 2020; Nguyen et al., 2022b). Notably, miR-539-5p exhibits potential significance in the pathogenesis of LPS-induced sepsis by selectively targeting *IRAK3*, suggesting its potential as a therapeutic target for treating LPS-induced sepsis (Hu and Miao, 2022). *SESN2*, a stress-inducible protein known as sestrin 2, plays a pivotal role in cellular stress responses and inflammation regulation. While SESN2 research has primarily focused on cancer and metabolic diseases (Lin et al., 2021; Xu et al., 2023), emerging evidence suggests its relevance to sepsis (Luo et al., 2020). Previous investigations have revealed that Sesn2-deficient mice exhibited impaired mitophagy, resulting in heightened inflammasome activation, and increased mortality in sepsis models (Kim et al., 2016). Multiple lines of evidence have substantiated the underlying mechanism, demonstrating that *SESN2* safeguards organismal and cellular homeostasis through the downregulation of reactive oxygen species accumulation and mammalian target of rapamycin protein kinase signaling (Peng et al., 2014). Additionally, *SESN2* suppresses DC ferroptosis in sepsis by downregulating the ATF4-CHOP-CHAC1 signaling pathway, suggesting antioxidative potential. In conclusion, *SESN2* contributes to immune response modulation and oxidative stress pathways central to sepsis pathophysiology. *CD59.* or Cluster of Differentiation 59, is a cell surface protein pivotal for inhibiting membrane attack complex formation and protecting against complement-mediated damage. Soluble *CD59* (sCD59) levels correlate with organ damage severity in sepsis patients, with elevated levels observed, particularly post-48 h intensive care unit admission (Ahmad et al., 2022). *CD59* has also emerged as a potential guardian against muscle tissue damage during sepsis (Wang et al., 2002), implicating its role in the complement system and immune response dysregulation contributing to sepsis pathogenesis. *SLC2A3* (Solute Carrier Family 2 Member 3), also referred to as *GLUT3* encodes a glucose transporter protein (Wu et al., 2021). While its direct role in PS remains uncertain, glucose metabolism and energy expenditure alterations are common in septic patients (Li and Mukhopadhyay, 2020; Ferreira et al., 2022). Plausible contributions of *SLC2A3* to metabolic adaptations during PS are conceivable. Further investigations are warranted to elucidate the potential relationship between *SLC2A3* and disease pathogenesis. *GYG1*, encoding Glycogenin-1, participates in glycogen synthesis (Fastman et al., 2022). Although its connection to PS remains unexplored, glucose metabolism and glycogen utilization disturbances are frequent in septic patients (Lu et al., 2022). *GYG1* deficiency contributes to glycogen storage diseases and polysaccharide myopathy (Visuttijai et al., 2020; Thomsen et al., 2022), hinting at its potential involvement in metabolic dysregulation associated with the disease. Extensive research is imperative to elucidate the specific involvement of *GYG1* in PS pathophysiology.

Despite our significant findings, there were limitations in this study The reliance on data from public databases for disease cohorts necessitates additional datasets to further validate the robustness of the diagnostic models. Incorporating *in vivo* data is essential for a comprehensive understanding of hub gene mechanisms in PS pathophysiology The integration of diverse datasets and experimental data holds promise for future advancements in PS research. Moreover, conducting a stratified analysis based on the sex, early or late stages of PS is meaningful.

## Conclusion

In this study, a comprehensive investigation of the expression patterns of CRGs in both PS samples and HC was performed. Leveraging the power of consensus clustering, we revealed distinct cuproptosis-associated clusters within the diseased samples, each characterized by unique immune profiles. Subsequently, we constructed a diagnostic model for PS based on the XGB algorithm and identified five specific genes. The model demonstrated robust performance, accurately classifying samples across qRT-PCR and independent validation datasets. Overall, our findings propose a novel diagnostic approach that not only elucidates the intricacies of disease heterogeneity but also provides insights into the immune microenvironment within PS.

## Data Availability

The datasets presented in this study can be found in online repositories. The names of the repository/repositories and accession number(s) can be found in the article/Supplementary Material.
