# Supplementary figures and images for: Deciphering the molecular classification of pediatric sepsis: integrating WGCNA and machine learning-based classification with immune signatures for the development of an advanced diagnostic model
Source: Front Genet. 2024 Jan 29;15:1294381. doi: 10.3389/fgene.2024.1294381 (PMC10859440; doi:10.3389/fgene.2024.1294381)

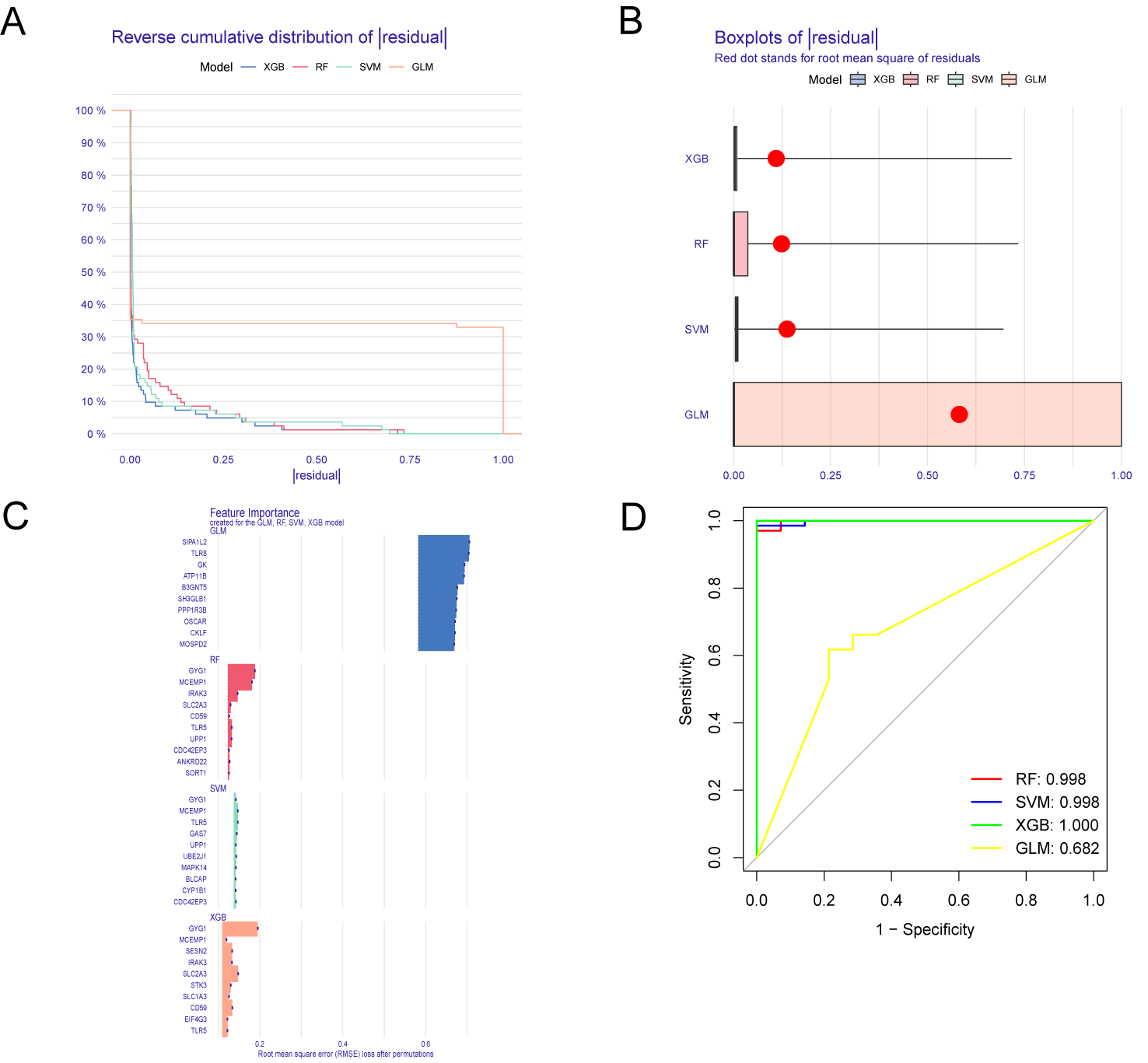

Supplement: Supplementary file 1 [file Image6.TIF]

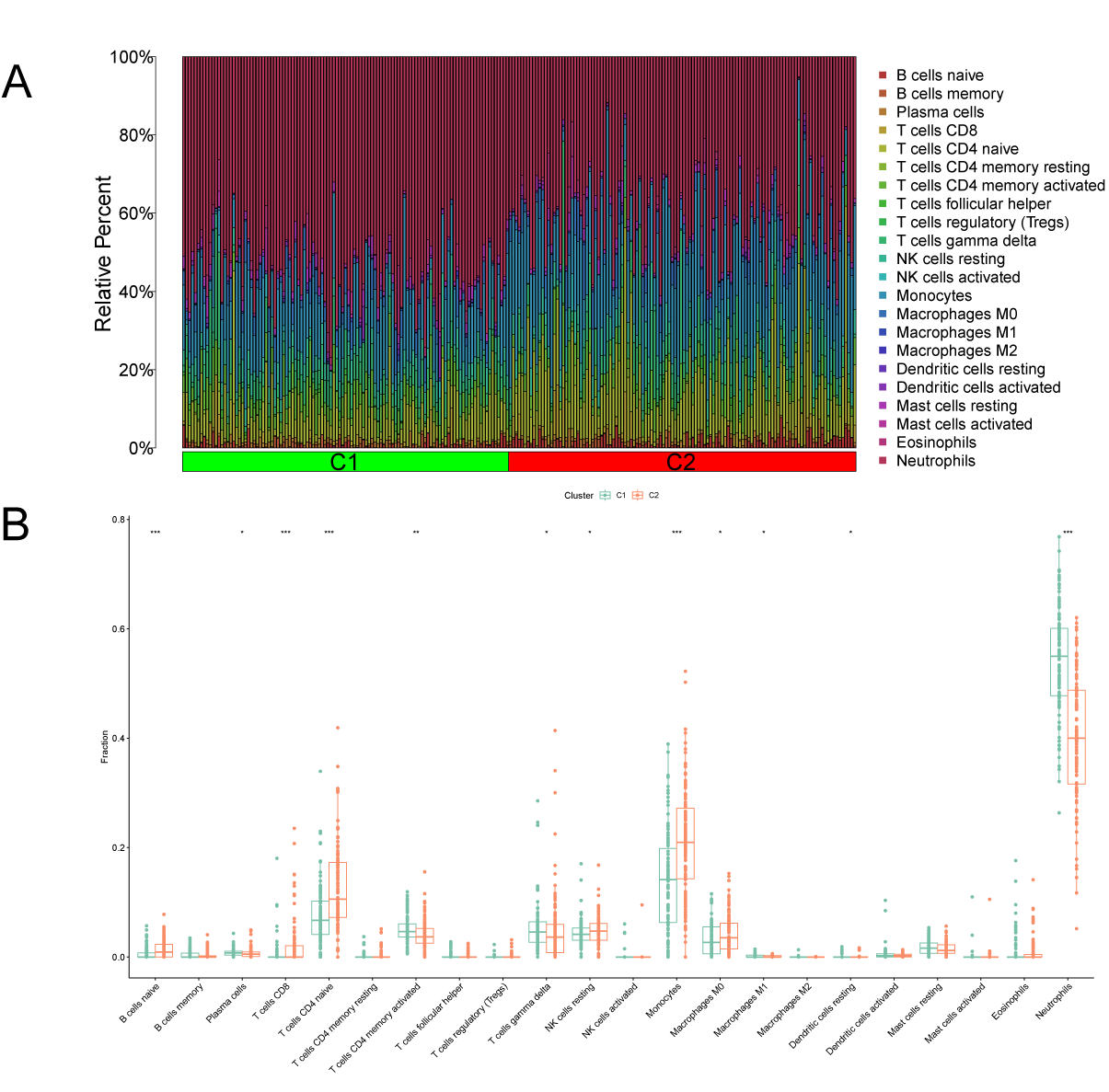

Supplement: Supplementary file 3 [file Image3.TIF]

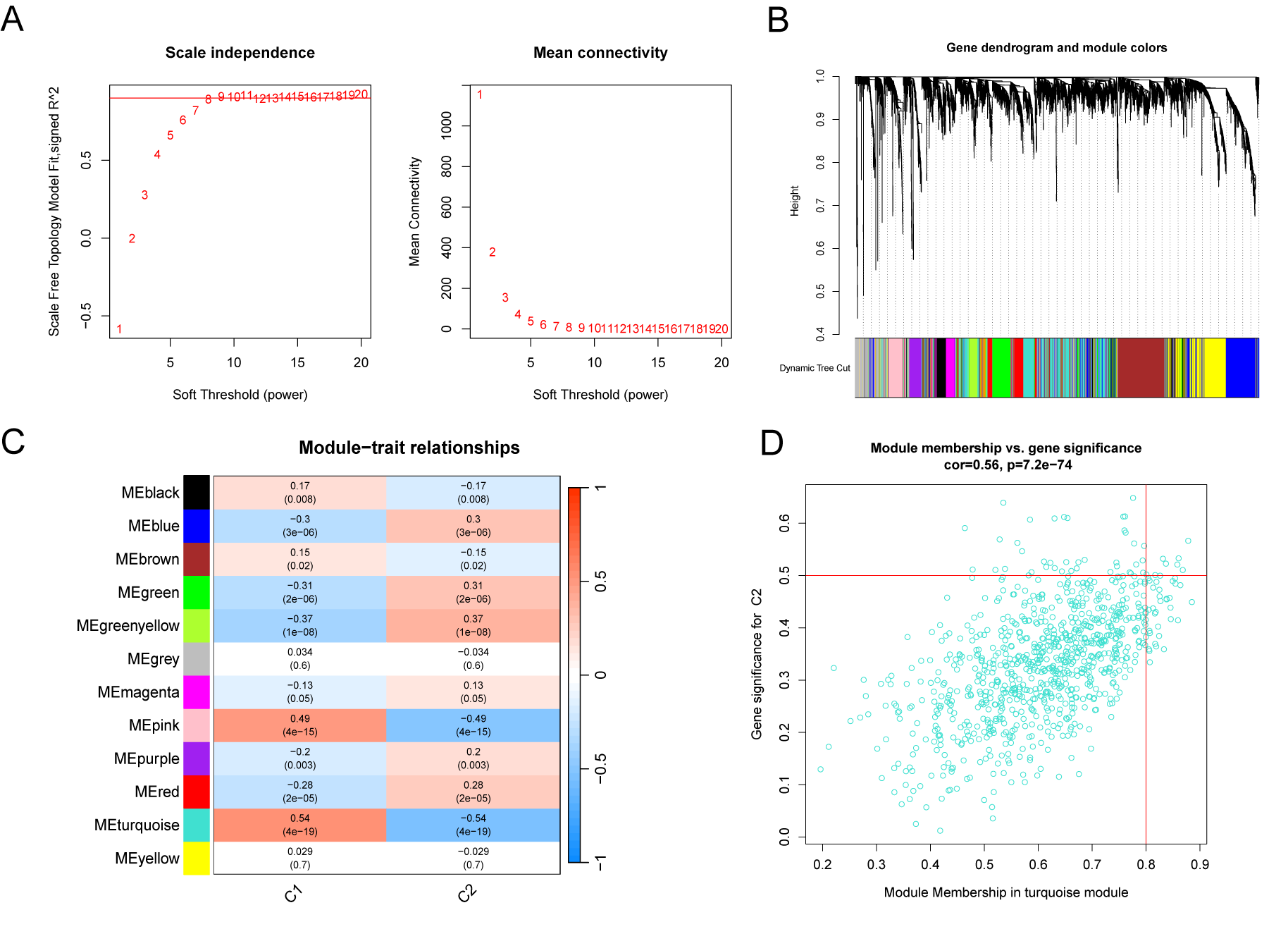

Supplement: Supplementary file 4 [file Image4.TIF]

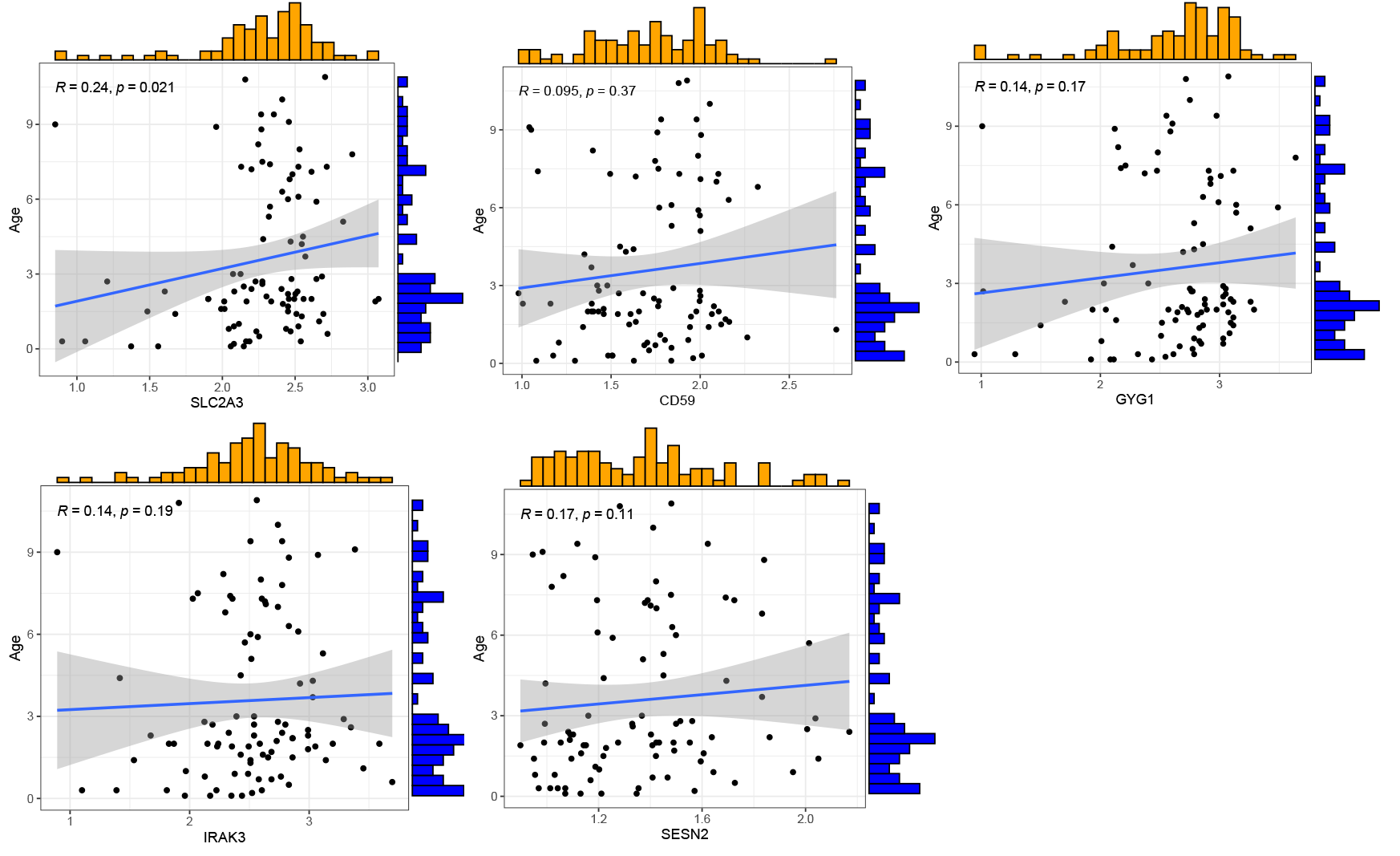

Supplement: Supplementary file 5 [file Image9.TIF]

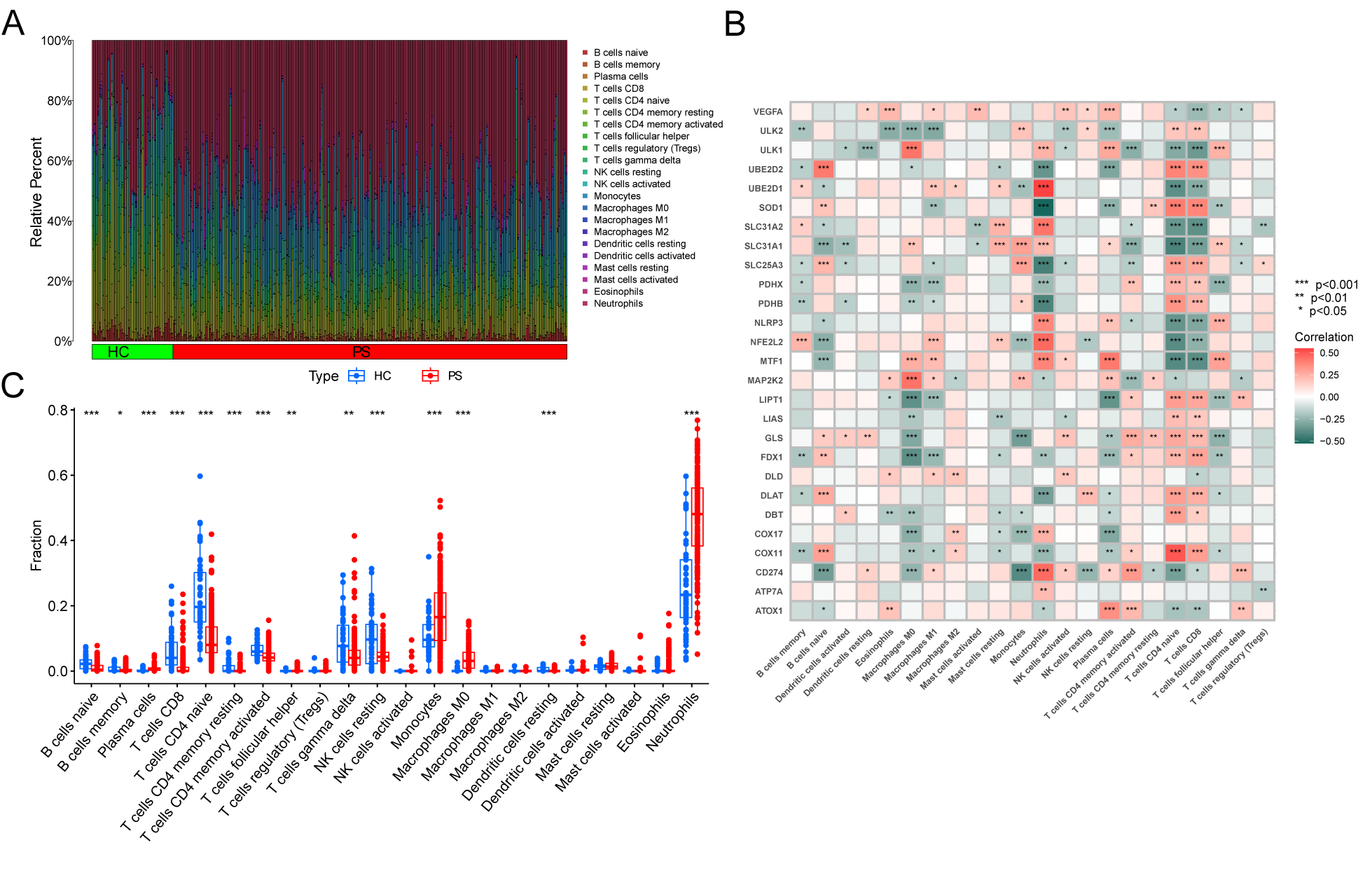

Supplement: Supplementary file 6 [file Image2.TIF]

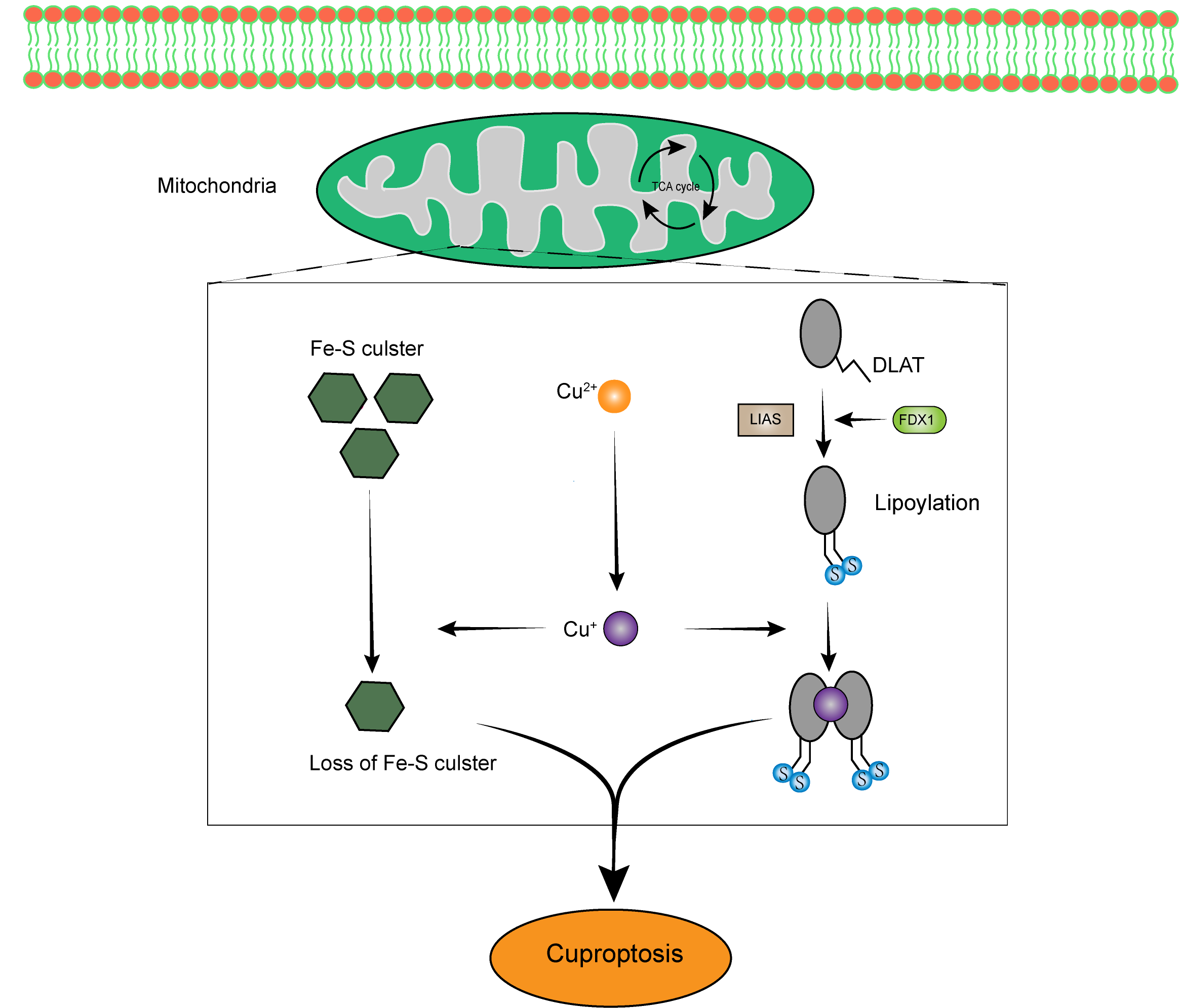

Supplement: Supplementary file 7 [file Image1.TIF]

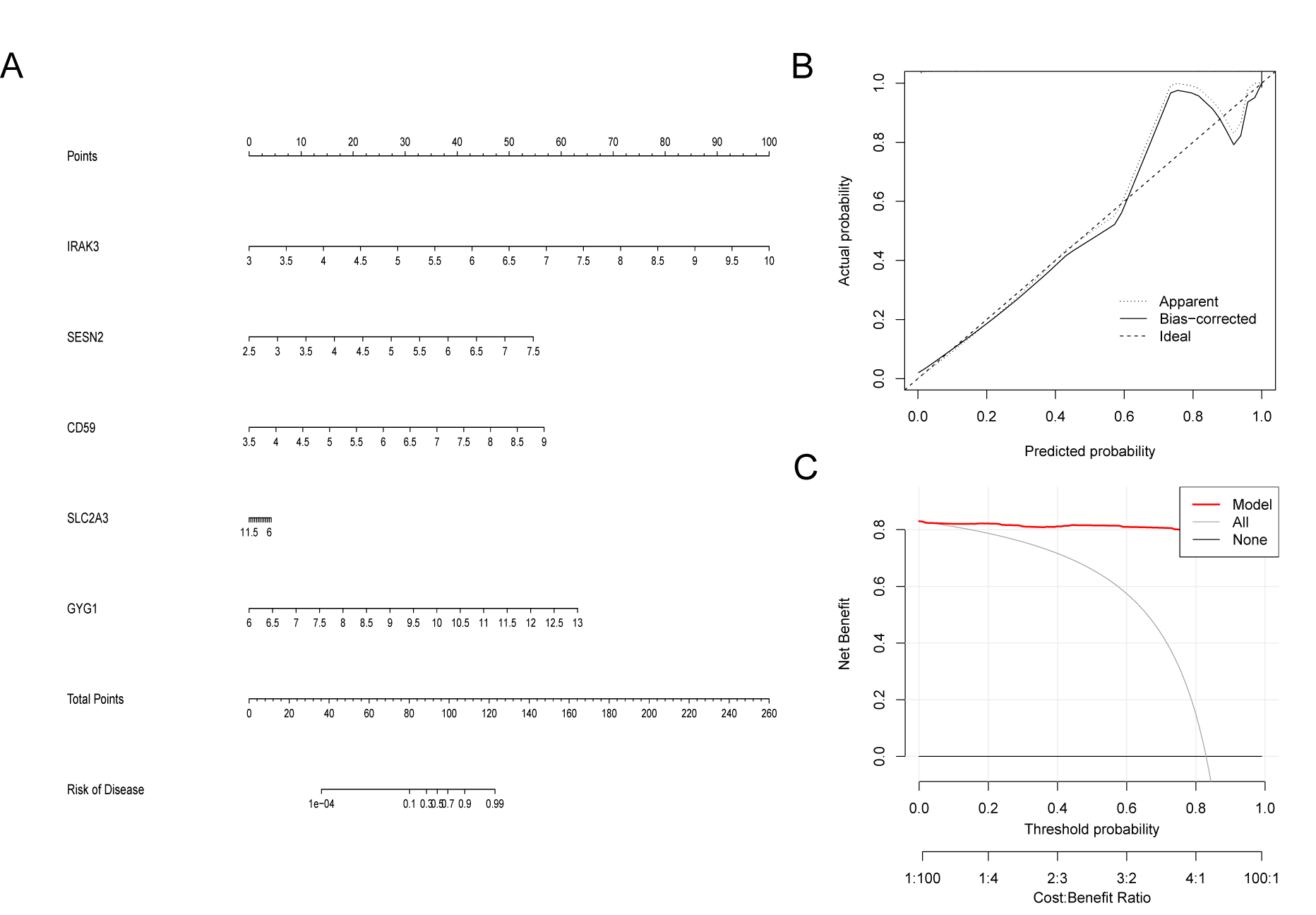

Supplement: Supplementary file 8 [file Image7.TIF]

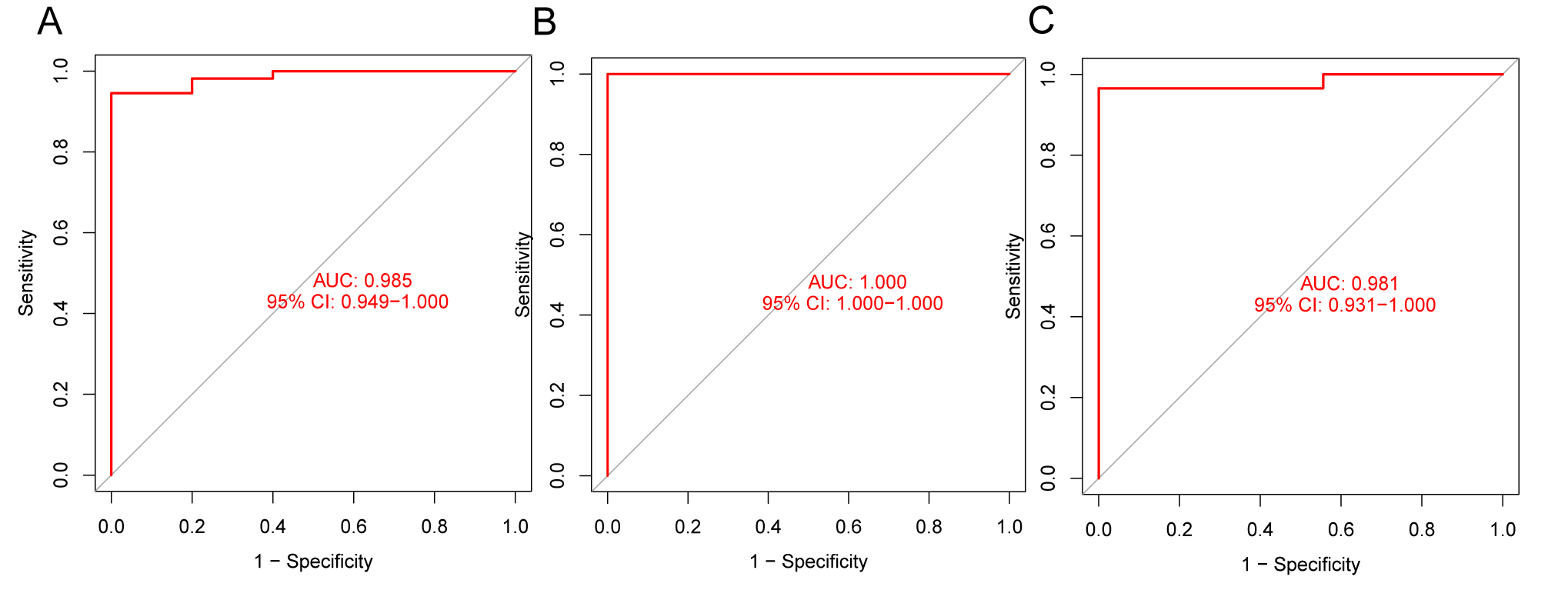

Supplement: Supplementary file 12 [file Image8.TIF]

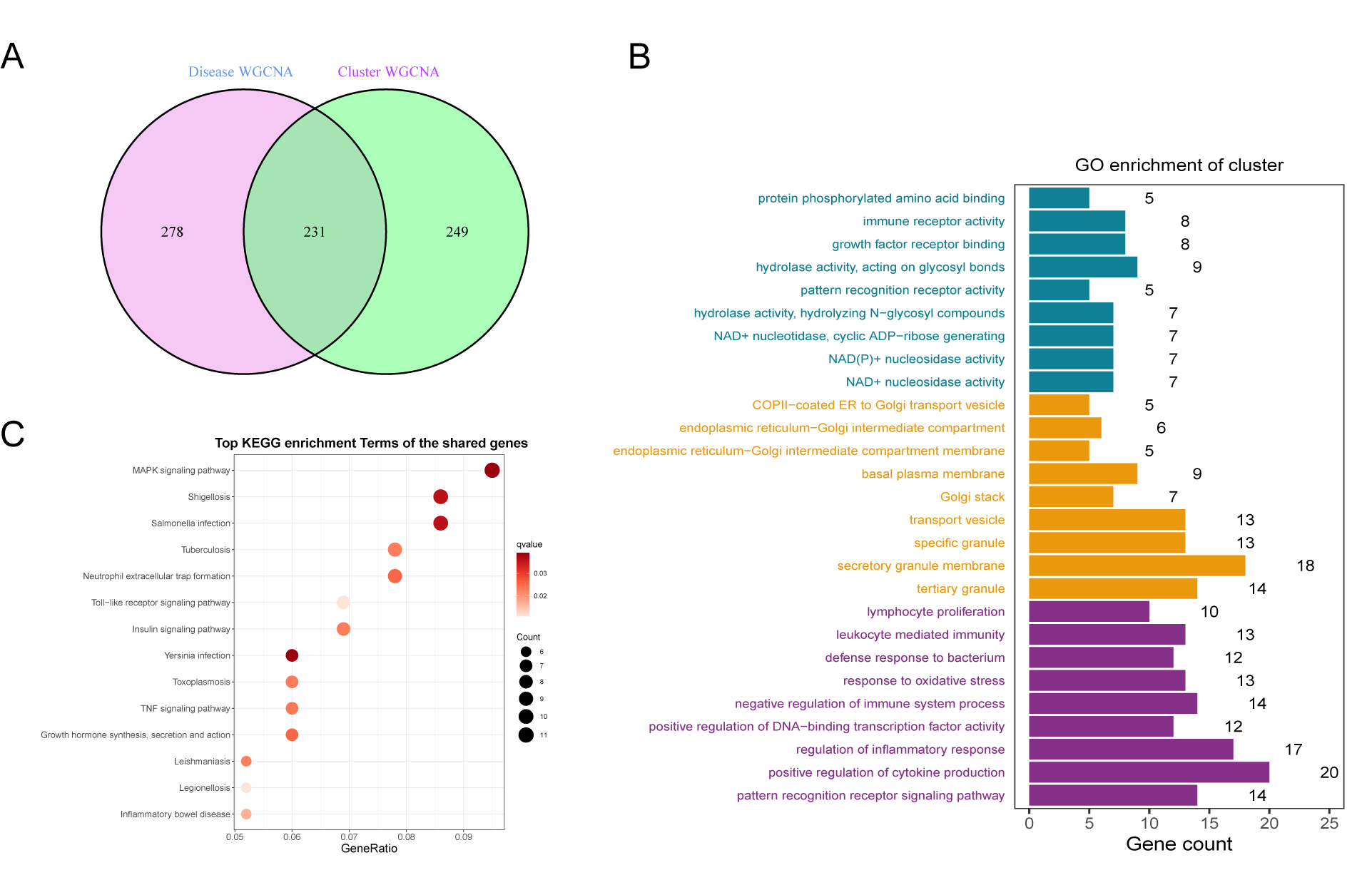

Supplement: Supplementary file 13 [file Image5.TIF]
