# Supplementary material for: Deciphering the molecular classification of pediatric sepsis: integrating WGCNA and machine learning-based classification with immune signatures for the development of an advanced diagnostic model
Source: Front Genet. 2024 Jan 29;15:1294381. doi: 10.3389/fgene.2024.1294381 (PMC10859440; doi:10.3389/fgene.2024.1294381)
Supplement: Supplementary file 2 [file Table1.DOCX]

Supplementary Table 1 Clinical information for HC and PS patients used in the qRT-PCR.

|  | Age | Sex | SOFA score | Diagnosis | Sample | Country |
| --- | --- | --- | --- | --- | --- | --- |
| PS 1 | 2 | Male | 3 | PS | Peripheral blood | China |
| PS 2 | 4 | Male | 3 | PS | Peripheral blood | China |
| PS 3 | 7 | Male | 4 | PS | Peripheral blood | China |
| PS 4 | 8 | Male | 5 | PS | Peripheral blood | China |
| PS 5 | 4 | Female | 2 | PS | Peripheral blood | China |
| PS 6 | 5 | Female | 4 | PS | Peripheral blood | China |
| PS 7 | 6 | Female | 4 | PS | Peripheral blood | China |
| PS 8 | 8 | Female | 5 | PS | Peripheral blood | China |
| HC 1 | 3 | Male | 0 | sterile inflammation | Peripheral blood | China |
| HC 2 | 7 | Male | 0 | sterile inflammation | Peripheral blood | China |
| HC 3 | 7 | Male | 0 | sterile inflammation | Peripheral blood | China |
| HC 4 | 8 | Male | 0 | sterile inflammation | Peripheral blood | China |
| HC 5 | 2 | Female | 0 | sterile inflammation | Peripheral blood | China |
| HC 6 | 3 | Female | 0 | sterile inflammation | Peripheral blood | China |
| HC 7 | 6 | Female | 0 | sterile inflammation | Peripheral blood | China |
| HC 8 | 8 | Female | 0 | sterile inflammation | Peripheral blood | China |
