# Supplementary material for: Deciphering the molecular classification of pediatric sepsis: integrating WGCNA and machine learning-based classification with immune signatures for the development of an advanced diagnostic model
Source: Front Genet. 2024 Jan 29;15:1294381. doi: 10.3389/fgene.2024.1294381 (PMC10859440; doi:10.3389/fgene.2024.1294381)
Supplement: Supplementary file 9 [file Table2.DOCX]

Supplementary Table 2 Primer sequences used in qRT-PCR.

| Gene | Primer direction | Sequence |
| --- | --- | --- |
| CD59 | Forward | CAGTGCTACAACTGTCCTAACC |
|  | Reverse | TGAGACACGCATCAAAATCAGAT |
| GYG1 | Forward | TGACACTAACCACAAACGATGC |
|  | Reverse | TAGATGAGCAGAATCGCCACT |
| IRAK3 | Forward | CTGCGGGATCTCCTTAGAGAA |
|  | Reverse | GCAGAGAAATTCCGAGGGCA |
| SLC2A3 | Forward | GCTGGGCATCGTTGTTGGA |
|  | Reverse | GCACTTTGTAGGATAGCAGGAAG |
| SESN2 | Forward | TCTTACCTGGTAGGCTCCCAC |
|  | Reverse | AGCAACTTGTTGATCTCGCTG |
| β-Actin | Forward | GTCATTCCAAATATGAGATGCGT |
|  | Reverse | GCTATCACCTCCCCTGTGTG |
