# Supplementary material for: Deciphering the molecular classification of pediatric sepsis: integrating WGCNA and machine learning-based classification with immune signatures for the development of an advanced diagnostic model
Source: Front Genet. 2024 Jan 29;15:1294381. doi: 10.3389/fgene.2024.1294381 (PMC10859440; doi:10.3389/fgene.2024.1294381)
Supplement: Supplementary file 10 [file Table3.DOCX]

Supplementary Table 3 The reagents used for ELISA.

| Protein | catalog number |
| --- | --- |
| CD59 | MM-51498H2 |
| GYG1 | MM-62659H2 |
| IRAK3 | MM-60593H2 |
| SLC2A3 | MM-62288H2 |
| SESN2 | MM-51154H2 |
