# Supplementary material for: Deciphering the molecular classification of pediatric sepsis: integrating WGCNA and machine learning-based classification with immune signatures for the development of an advanced diagnostic model
Source: Front Genet. 2024 Jan 29;15:1294381. doi: 10.3389/fgene.2024.1294381 (PMC10859440; doi:10.3389/fgene.2024.1294381)
Supplement: Supplementary file 11 [file Table4.DOCX]

Supplementary Table 4 The role of 59 CRGs in the cuproptosis pathway.

| Gene | Description |
| --- | --- |
| AOC3 | AOC3 encodes a copper-dependent enzyme involved in the metabolism of amines. Its role in cuproptosis may be linked to its participation in oxidative reactions. |
| ATOX1 | ATOX1 is a crucial copper-binding protein involved in copper transport and regulation. Upon binding with copper ions, it guides copper to the appropriate cellular locations to perform its biological functions |
| ATP7A | Encode copper transport proteins located on the cell membrane |
| ATP7B | Encode copper transport proteins located on the cell membrane |
| CCS | SOD1 is a copper-containing enzyme that clears superoxide radicals within the cell. CCS helps SOD1 in its proper folding and activity by providing copper ions. This is crucial for maintaining the redox balance within the cell and preventing oxidative damage. |
| CD274 | CD274, also known as PD-L1, is not directly associated with copper metabolism. However, it may play a role in immune regulation, and its involvement in cuproptosis might be related to immune response modulation. |
| CDKN2A | CDKN2A is a tumor suppressor gene that regulates the cell cycle. Its role in cuproptosis could be associated with its impact on cell proliferation and apoptosis. |
| COAS | COAS is likely involved in delivering copper to cytochrome c oxidase, an essential enzyme in the mitochondrial respiratory chain. |
| COX11 | COX11 involved in copper delivery to cytochrome c oxidase and are crucial for mitochondrial function. |
| COX17 | COX17 involved in copper delivery to cytochrome c oxidase and are crucial for mitochondrial function. |
| CP | CP is a copper-carrying protein. Its role in cuproptosis might be linked to its functions in oxidative stress regulation. |
| DBH | In cuproptosis, alterations in DBH activity could impact neurotransmitter synthesis and contribute to neuronal dysfunction. |
| DBT | DBT is a component of the pyruvate dehydrogenase complex, a key player in energy metabolism. It contains lipoic acid, which forms a complex with copper. Changes in copper availability or metabolism may influence the function of DBT, affecting cellular energy balance. |
| DLAT | DLAT is another component of the pyruvate dehydrogenase complex. It interacts with lipoic acid and is involved in the conversion of pyruvate to acetyl-CoA. Like DBT, DLAT's function can be influenced by copper, and disruptions in copper homeostasis may impact its role in energy metabolism. |
| DLD | DLD is the third component of the pyruvate dehydrogenase complex. It plays a crucial role in the regeneration of lipoic acid. As with DBT and DLAT, DLD's function is intertwined with copper, and disturbances in copper levels may affect its activity, leading to cellular dysfunction. |
| DLST | DLST is part of the α-ketoglutarate dehydrogenase complex, which is involved in the tricarboxylic acid (TCA) cycle. Copper is known to influence enzymes in the TCA cycle, and changes in DLST function due to altered copper levels could impact cellular metabolism and contribute to cuproptosis. |
| FDX1 | FDX1 have emerged as pivotal orchestrators during copper ionophore-induced cell death |
| GCSH | GCSH is among the four known mammalian enzymes that can undergo protein lipoylation. Therefore, GCSH is an essential target involved in the copper death process. |
| GLS | Plays a role in glutamine metabolism. Alterations in glutamine metabolism can impact cellular redox balance and contribute to oxidative stress, a hallmark of cuproptosis. |
| LIAS | Involved in iron-sulfur cluster biogenesis. Copper and iron metabolism are interconnected, and disturbances in one can affect the other, potentially influencing cuproptosis. |
| LIPIT1 | Lipid metabolism genes. Changes in lipid metabolism can impact cell survival and may contribute to cell death processes. |
| LIPT1 | Lipid metabolism genes. Changes in lipid metabolism can impact cell survival and may contribute to cell death processes. |
| LIPT2 | Lipid metabolism genes. Changes in lipid metabolism can impact cell survival and may contribute to cell death processes. |
| LOXL2 | A lysyl oxidase involved in extracellular matrix remodeling. Dysregulation may affect tissue integrity and contribute to pathological conditions, potentially including cuproptosis. |
| MAP2K1 | Components of the MAPK signaling pathway. MAPK pathways can be activated in response to stress and may contribute to cell death mechanisms. |
| MAP2K2 | Components of the MAPK signaling pathway. MAPK pathways can be activated in response to stress and may contribute to cell death mechanisms. |
| MTCO2P12 | Pseudogene related to mitochondrial cytochrome c oxidase subunit II. Mitochondrial dysfunction is often associated with cuproptosis. |
| MTF1 | A transcription factor involved in metal homeostasis. It may regulate copper-related genes and impact cuproptosis susceptibility. |
| NFE2L2 | Namely Nrf2, a transcription factor that regulates antioxidant response element (ARE)-mediated genes. NFE2L2 activation can influence cellular redox status. |
| NLRP3 | Part of the NLRP3 inflammasome, which can be activated in response to cellular stress and contribute to inflammatory processes associated with cuproptosis. |
| PDE38 | Phosphodiesterase involved in cAMP metabolism. Cellular signaling pathways influenced by PDE38 may be implicated in cuproptosis. |
| PDHA1 | Genes related to pyruvate metabolism. Changes in pyruvate metabolism can influence cellular energetics and contribute to cell death. |
| PDHB | Genes related to pyruvate metabolism. Changes in pyruvate metabolism can influence cellular energetics and contribute to cell death. |
| PDK1 | Genes related to pyruvate metabolism. Changes in pyruvate metabolism can influence cellular energetics and contribute to cell death. |
| SCO1 | Involved in the assembly of cytochrome c oxidase. Perturbations in mitochondrial function may contribute to cuproptosis. |
| SLC25A3 | Mitochondrial phosphate carrier. Mitochondrial transporters are critical for cellular energetics and may be involved in cuproptosis. |
| SLC31A1 | Copper transporters. Essential for copper homeostasis and their dysregulation can influence cuproptosis susceptibility. |
| SLC31A2 | Copper transporters. Essential for copper homeostasis and their dysregulation can influence cuproptosis susceptibility. |
| SOD1 | Superoxide dismutase. Involved in antioxidant defense. Dysregulation may impact cellular redox balance. |
| TYR | Tyrosinase. Involved in melanin synthesis. Its role in cuproptosis may involve pigment-related processes. |
| UBE2D1 | Ubiquitin-conjugating enzymes. Involved in protein degradation pathways that may be implicated in cuproptosis. |
| UBE2D2 | Ubiquitin-conjugating enzymes. Involved in protein degradation pathways that may be implicated in cuproptosis. |
| UBE2D3 | Ubiquitin-conjugating enzymes. Involved in protein degradation pathways that may be implicated in cuproptosis. |
| UBE2D4 | Ubiquitin-conjugating enzymes. Involved in protein degradation pathways that may be implicated in cuproptosis. |
| ULK1 | Unc-51-like autophagy activating kinases. Autophagy pathways can be influenced by copper and may play a role in cuproptosis. |
| ULK2 | Unc-51-like autophagy activating kinases. Autophagy pathways can be influenced by copper and may play a role in cuproptosis. |
| VEGFA | Vascular endothelial growth factor A. Its role in angiogenesis and inflammation may contribute to cuproptosis-related processes. |
| ARFGEF1-DT | unknown |
| LINC03085 | unknown |
| SMAD3-AS1 | unknown |
| C6ORF136 | unknown |
| H2BC6-AS1 | unknown |
| H1-10-AS1 | unknown |
| FOXD2-AS1 | unknown |
| LINC02154 | unknown |
| LASTR | unknown |
| HSPD1 | unknown |
| PDHX | unknown |
| H3C1 | unknown |
